# Non-Invasive Sampling in the Aspect of African Swine Fever Detection—A Risk to Accurate Diagnosis

**DOI:** 10.3390/v14081756

**Published:** 2022-08-11

**Authors:** Marek Walczak, Anna Szczotka-Bochniarz, Jacek Żmudzki, Małgorzata Juszkiewicz, Krzesimir Szymankiewicz, Krzysztof Niemczuk, Daniel Pérez-Núñez, Lihong Liu, Yolanda Revilla

**Affiliations:** 1 National Veterinary Research Institute, 57 Partyzantów Avenue, 24-100 Pulawy, Poland; 2 Centro de Biología Molecular Severo Ochoa, CSIC-UAM, Microbes in Health and Welfare Department, c/Nicolás Cabrera 1, 28049 Madrid, Spain; 3 National Veterinary Institute, SE-756 51 Uppsala, Sweden

**Keywords:** ASF, ASFV, sampling, detection, diagnosis

## Abstract

African swine fever remains one of the most economically important and dangerous diseases of the *Suidae* family. Until now, neither a safe vaccine nor a treatment against ASF has been available, which is why prevention of the disease involves biosecurity measures and early recognition based on accurate diagnosis. Nowadays, different strategies for ASF detection are discussed to reduce both animal suffering and the costs of ASF surveillance. This article aims to indicate the risk, with regard to non-invasive sampling, for the detection of ASFV. In this study, we analyzed data from three independent animal trials, in the framework of the detection of positive samples in different matrices (blood, sera, oral and rectal swabs) collected from nineteen domestic pigs infected with similar doses but under different scenarios, including different ASFV strains or routes of infection. Genetic material of ASFV was found in all matrices, but detection occurred earlier in the blood samples than in the oral and the rectal swabs. Furthermore, analyses revealed that at relevant sampling timepoints, PCR-positive blood samples were detected more frequently and reached higher percentages (up to 100% during fever) than oral and rectal swabs. Moreover, mean Ct values in blood samples collected from animals infected with virulent strains were significantly lower than in oral and rectal swabs, ensuring a higher probability of ASFV detection. High Ct values and occasional shedding in all tested matrices, in the cases of animals infected by an attenuated ASFV-strain, showed that blood sampling may be necessary to confirm the presence of anti-ASFV antibodies in sera. This study showed that during veterinary surveillance, blood sampling (for both PCR and serological analyses) is essential for the accurate diagnosis of ASF and provides the highest probability of detection of the disease.

## 1. Introduction

The proper diagnosis of infectious diseases plays a crucial role in early recognition and the prevention of further spread. It is particularly important when an outbreak of potential disease can affect animals severely and can have an enormous socio-economic impact on people. However, the invasive screening for infectious diseases might be time-consuming and costly, while also being a source of animal stress. Nowadays, society is becoming more aware of “animal welfare”, a term which has been briefly defined by Broom as a state of the animal regarding its attempts to cope with its environment [1,2]. One of the key points of maintaining animal welfare is to avoid animal pain induced by inappropriate management [3]. Blood sampling is a basic method used in veterinary surveillance, however, it may be harmful to animals and labor intensive [4]. Animal stress and pain may be reduced, depending on the blood sampling method [3,5], but this is not completely free of adverse effects. Due to these reasons, non-invasive strategies for surveillance sampling have been discussed by several authors [4,6,7,8]. In light of this, new approaches to sample collection during veterinary surveillance are necessary and justified. Nonetheless, without knowing a particular pathogen’s nature, alternative methods of sampling may not be efficient for the proper diagnosis of the disease, which may lead to drastic consequences for both animals and humans.

One of the most economically important diseases of swine is African swine fever (ASF), caused by the African swine fever virus (ASFV). The virus is responsible for the current ASF epidemic in Europe and Asia and it has caused enormous economic losses, paralyzing the world’s trade of pork [9]. In China, for instance, between 2018 and 2019, ASF led to a 0.78% gross domestic product (GDP) loss, which was equal to USD 111.2 billion [10]. In 2021, Vietnam forecast a 0.36% to 1.8% GDP ASF-related reduction (equal to USD 0.88–4.40 billion), depending on the scenario [11]. It is noteworthy that the outbreak of ASF did not only affect animals, but also caused dramatic social impact (i.e., loss of jobs) in the relevant food production sectors.

ASFV is a large DNA virus, infectious for the *Suidae* family [12]. The virus enters the body via pharyngeal mucosa and reaches the nearest lymphatic organs (tonsils and submandibular lymph nodes), from where the virus spreads systemically within the blood. Shortly after, ASFV can be found in almost all tissues [13]. The presence of the virus can also be confirmed in oral fluid, faces and urine [14,15]. During infection, the virus targets myeloid line-cells, mainly monocytes and macrophages, which leads to devastating inflammation processes [16,17]. ASF-affected animals present a high mortality rate, up to 100%. Moreover, there are no commercially available and safe vaccines against ASF. Combatting the disease is mainly based on biosecurity measures and early recognition of the pathogen among wild boars or domestic pigs [18]. Strategies of surveillance of the disease include passive and active surveillance, with the use of the screening of blood or internal organ samples [19]. Detection of ASFV may be carried out through the detection of antigens or through the presence of specific anti-ASFV antibodies. Detection of the antigen includes classic virus isolation, ELISA tests and conventional PCR tests, however, real-time PCR has become a gold standard in ASFV detection, ensuring high levels of sensitivity and specificity [20,21].

Recent and previously published studies have proven that the detection of genetic material of ASFV is possible in most of the studied matrices, as well as in the matrices suitable for non-invasive surveillance. Several authors have reported that nasal and oral swabs may be found to be PCR-positive in cases of parenteral infection, intramuscular infection or in in-contact pigs [15,22,23]. The presence of ASFV DNA was also confirmed in oral and rectal swabs by Olesen et al. [24]. Goonewardene et al. showed that an aggregated sample of oral fluid may be suitable for ASFV surveillance [7]. As most of these studies were focused mainly on the characterization of disease kinetics, positive results regarding detection of ASFV DNA in the different matrices may suggest that employing non-invasive sampling may be sufficient for the accurate diagnosis of ASF. Unfortunately, none of the studies highlight the risk regarding a low virus titer, the frequency of positive samples or the time of sampling accuracy.

Published studies have proven that the detection ASFV DNA in blood is reliable and usually occurs earlier than in other matrices. However, shedding patterns and viral loads may differ, depending on the matrices, the virus isolates or the route of infection [22,23,25].

Successful detection of the pathogen depends not only on the choice of a highly specific and sensitive method, but also on the choosing of proper samples and an accurate time of sampling. This study gathers data from three independent animal trials and aims to present risks related to proper sample selection, in the framework of the accurate detection of ASF under different scenarios, including different ASFV strains or routes of infection.

## 2. Material and Methods

### 2.1. Experimental Settings and Ethics

A total of nineteen domestic pigs of the Danbred × Duroc strain, both sexes, 6-weeks old, were infected with different ASFV strains in three independent trials. The number of animals, ASFV strain characteristics, route of infection and doses are summarized in Table 1. Disease kinetics (rectal temperature and survival rate) are presented in Supplementary Figure S1.

The animals were kept in the BSL3 (Biosafety Level-3) animal facility at the National Veterinary Research Institute (Puławy, Poland), with feed and water ad libitum. Before infection, the health status of all animals was evaluated through veterinary examination and confirmed to be free of ASFV by using a Virotype^®^Real-time PCR kit (Qiagen, Hilden, Germany). The animal experiment was approved by the local Ethical Commission for Animal Experiments in Lublin (the approval number: 56/2020 Trial 1 and Trial 3, and 145/2018 Trial 2). All procedures, including euthanasia, were done according to actual legal regulations.

### 2.2. Viruses

A virulent Pol18_28298_O111 (Pol18) strain, belonging to genotype II of ASFV, was derived from the spleen of naturally infected pigs (outbreak No. 111, Chełm district, Lubelskie vovoidship, May 2018) [26]. Porcine alveolar macrophages (PAM) and RPMI medium (Gibco, Thermo Fisher Scientific, Waltham, MA, USA) supplemented with 10% fetal bovine serum (FBS) were used for virus propagation. Diluted stock to the dose 10^3^ HAD_50_/mL phosphate-buffered saline (PBS) was intended for oronasal infection [14]. Strain Arm07/CBM/c2 (Arm07) was derived from an original stock of Armenia 07 (genotype II) in the Centro de Biología Molecular Severo Ochoa, CSIC-UAM (Madrid, Spain) [27] and kindly provided by the EU Reference Laboratory for ASF (CISA-INIA, Valdeolmos, Spain) together with attenuated NH/P68 (non-hemadsorbing virus Portugal 68) strain (genotype I). The field-attenuated ASFV strain NH/P68 [28] and the field-virulent strain Armenia/07/CBM/c2 were grown in porcine alveolar macrophages (PAM) (cultured in Dulbecco’s modified Eagle’s medium (DMEM) supplemented with 10% pig serum (Sigma Aldrich, St. Louis, MO, USA)) and titrated by plaque assay on COS-1 cells or by hemadsorption with erythrocytes in PAM, respectively, as previously described [27,29]. Stocks of both viruses were diluted to obtain dose 10^3^ TCID_50_/mL and intended for intramuscular infection.

### 2.3. Fever Records and Samples

Rectal temperature was recorded on a daily basis during all three trials. The fever threshold was set to 40 °C.

Oral and rectal swabs were collected on a daily basis, within 0–8 days post-infection (dpi) during T1, 0–21 dpi during T2 and 0–28 dpi during T3. After sample collection, swabs were placed into tubes containing 4 mL (T2) or 1 mL (T1 and T3) of PBS, then incubated in room temperature for 10 min and vortexed. An aliquot of 200µL of each sample was used for DNA extraction.

Blood was collected into plastic MLVacuCol tubes (Medlab, Raszyn, Poland) containing K2-EDTA. During T1 and T3, animals were sampled every other day; in T3, beginning from 0 to 14 dpi, then at 21 and 28 dpi. During T2, blood was collected at −7, 0, 1 and 4 dpi, then at least two times a week or daily, whenever first clinical signs (i.e., fever) were recorded.

Blood was diluted 1:10 in PBS (*v*/*v*), 200 µL of each sample intended for DNA extraction.

### 2.4. DNA Extraction and Real-Time PCR Analysis

Manual column DNA extraction was carried out in accordance with the Qiagen DNA Mini Kit protocol (Qiagen, Hilden, Germany). A Virotype ASFV PCR kit (Indical Bioscience Gmbh, Leipzig, Germany) was used to conduct a real-time PCR assay, in a Rotor-GeneQ thermocycler (Qiagen, Hilden, Germany), according to the manufacturer’s instructions. A commercial, positive control from an ASFV PCR kit (Indical Bioscience Gmbh, Leipzig, Germany) was used and set to approximately 30 Ct to ensure maximal comparability between viral loads, in each run.

### 2.5. Antibody Detection and Titration

Specific anti-ASF antibodies were detected by an indirect immunoperoxidase assay (IPT) described by the EU Reference Laboratory for ASF (CISA-INIA, Valdeolmos, Spain) using the standard operating procedure (SOP/CISA/ASF/IPT/1). Sera collected on the day of euthanasia (T3) or the last blood-sampling timepoint (T1 and T2) before the death of the animals were used for antibody titration by IPT. Titration was done in 2-fold (T2) or 10-fold (T1 and T3) serial dilutions.

### 2.6. Blood vs. Swabs

For the purpose of comparison, real-time PCR results were obtained from relevant sampling timepoints from both blood and swabs (rectal and oral), which were analyzed from the beginning of the experiment and during the fever period.

### 2.7. Statistical Analysis

Statistically significant differences between groups were calculated by a one-way analysis of variance (ANOVA) with a Dunnett’s correction or the Kruskal–Wallis test, in GraphPad Prism 8.4.2 (GraphPad Software, San Diego, CA, USA). Means are presented with standard deviations (±SD). Difference at *p* < 0.05 was considered significant.

## 3. Results

### 3.1. Overall Detection of Positive Animals during Trials

All collected samples were tested and the presence of ASFV DNA (in blood, oral and rectal swabs) or specific anti-ASFV antibodies in sera are shown in Table 2.

Based on results, the majority of the infected animals could be positively diagnosed by PCR (when tested regularly from the first day of infection) or serology (in case of the attenuated strain infection). In contrast to the animals infected by the virulent strains, where only two pigs were found seropositive (1/T1, first detection in 7 dpi and 1/T2, first detection in 13 dpi), specific anti-ASFV antibodies were found in all animals from T3 starting from 11–12 dpi. Moreover, in this group, antibody response was about 10-fold stronger than in T1 and T2 (Table 2).

Despite the daily sampling of oral and rectal swabs, we identified cases of single detections of ASFV DNA during the whole trial period (T1 0–8 dpi, T2 0–21 dpi, T3 0–28 dpi). A single detection of viral DNA in oral swabs was found in three animals (1/T1, 1/T2 and 1/T3). In the case of rectal swabs, we found six instances of a single detection of the virus’ genetic material (1/T1, 3/T2, 2/T3). Individual results of ASFV DNA detection in oral, rectal and blood samples are presented in the supplementary Table S1.

**Figure 1:**
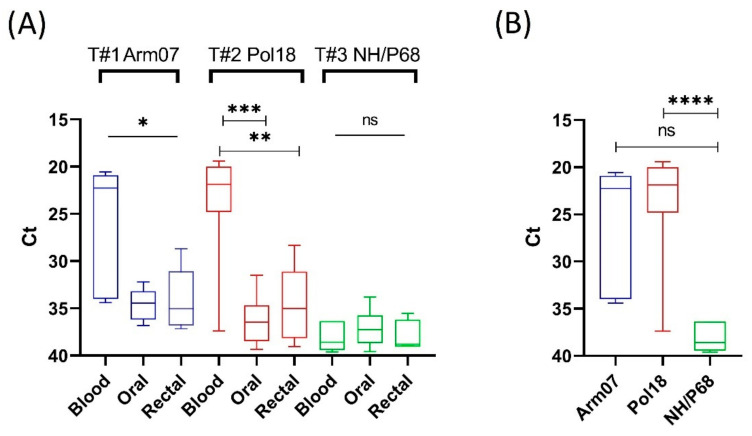
(**A**) Statistical analysis of mean Ct values in different matrices within respective trial groups. (**B**) Statistical analysis of mean Ct value recorded in blood between virulent and attenuated strains; * statistically significant (T1, blood vs. oral and rectal swabs, *p* = 0.0335 and *p* = 0.0450, respectively), ** statistically significant (T2, blood vs. rectal swabs, *p* = 0.0011), *** statistically significant (T2, blood vs. oral swabs, *p* < 0.0001), **** statistically significant (Pol18 vs. NH/P68, *p* = 0.004), ns—not significant. The boxes represent the 50% between the 25 and 75% quartiles. The line inside the box indicates the median. The top and bottom lines denote maximum and minimum values.

Importantly, the viral load between blood, rectal and oral swabs differed significantly in animals infected with virulent strains (T1 and T2). The mean Ct value was significantly lower in blood, while in rectal and oral swabs the value remained at approximately 35.0 Ct, close to the real-time PCR threshold (Figure 1A). Mean Ct values in all matrices from T3 were similarly high, ranging from 36.6 (±2.4) to 38.0 (±1.4). The mean Ct value in blood was noticeably lower in groups infected with virulent strains and differed significantly between T2 and T3 (*p* = 0.004), but not between T1 and T3 (*p* = 0.0504) (Figure 1B).

### 3.2. Virus Shedding and Fever

The virus’ shedding pattern differed, depending on the disease dynamics caused by different strains or routes of infection. In animals infected intramuscularly (T1) with the virulent strain, viral DNA could be detected earlier than in animals infected via the intranasal route (T2) or infected with the attenuated NH/P68 strain (T3). In addition, almost all recorded mean latent periods were shorter in blood than in oral and rectal swabs, except the T3, where ASFV DNA was detected first in rectal swabs (Table 3).

In T1 and T2, fever was detectable shortly after viremia detection. A 1-day delay in fever, regarding viremia, was noticed in the cases of 4/6 animals from T1 and 3/7 animals from T2, while in T3, fever was detectable occasionally and, mostly, was not correlated to detectable viremia. Results for mean latent periods and mean incubation periods (first fever) are summarized in Table 3.

**Figure 2:**
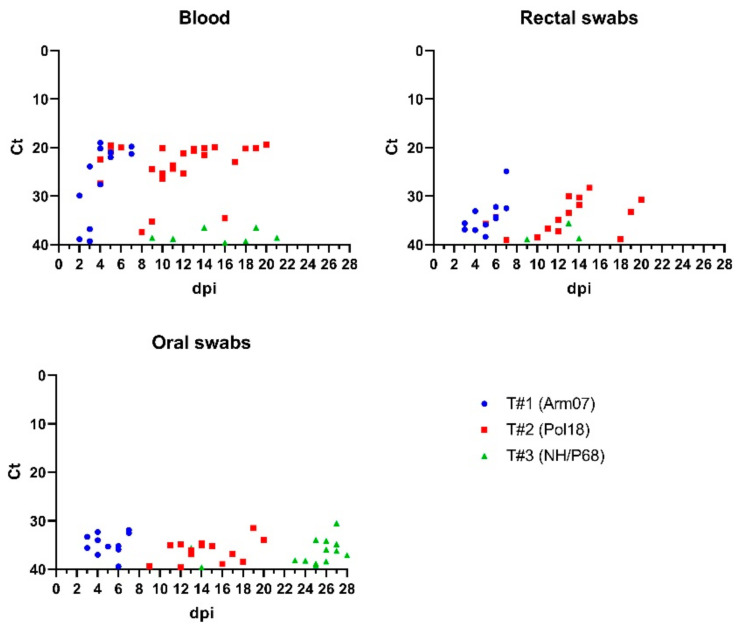
The shedding pattern of viral DNA in different matrices analyzed during animal trials. Lower Ct values in blood in animals infected with virulent strains and high Ct values accompanied by occasional shedding in groups infected with attenuated strain could be seen; Ct—cycle threshold of qPCR, dpi—day post infection.

In the virulent strains, ASFV DNA was detectable from the first day of viremia until animal death, while in animals infected with the attenuated NH/P68 strain, viral DNA in blood was occasionally detectable. Despite the longest trial period and sampling in T3 (0–28 dpi), single detection in blood occurred in two pigs, while two others remained negative for ASFV DNA. One case of single detection of virus’ genetic material in blood was identified in T1, however the presence of ASFV DNA was further confirmed post mortem (Table S1). The shedding pattern is presented in Figure 2.

### 3.3. Blood vs. Swabs Comparison

At the same sampling timepoints during the whole period of the experiments, PCR-positive blood samples reached higher percentages (ranging from 11.1% to 68.8%) than PCR-positive samples of oral and rectal swabs (6.3−37.5% and 4.8−37.5%, respectively). The number of relevant sampling timepoints depended on the disease dynamics. The number was highest in the group infected with the attenuated NH/P68 strain. Despite the highest number of relevant sampling timepoints, shedding of the virus in this group was occasional and led to the lowest percentage of PCR-positive samples (Table 4).

During the fever period, the percentages of PCR-positive samples were the highest in animals infected with the virulent strains, in blood reaching 100%, in oral and rectal swab samples ranging from 65.0 to 80%. In contrast to T1 and T2, fever in T3 was occasional and not correlated with shedding. Therefore, the percentages of PCR-positive samples reached much lower rates, even 0% in the case of oral or rectal swabs (Table 5).

## 4. Discussion

The detectability of an animal’s virus pathogens in different samples collected during veterinary surveillance depends on many factors.

First and foremost, are the characteristics of the virus, such as host and targeted cells. ASFV belongs to viruses which target myeloid-line cells existing predominantly in blood or lymphatic organs [30]. Therefore, it is natural that observable shedding of the virus, in such matrices as saliva, feces or urine, may occur at lower levels (higher Ct) than in blood. In the present study, shedding of the virus (at different levels) was confirmed in all analyzed matrices, which is in accordance with previously published studies [23,24,25]. It is noteworthy that our results, regarding sporadic shedding and high Ct values in animals infected with attenuated strains of ASFV (NH/P68), are similar to the results presented by Kosowska et al. and Gallardo et al. [25,31], and the viral load in blood is much lower compared with the virulent strains.

Second, is the choosing of an adequate method (ensuring a high level of specificity and sensitivity) and respective samples. The real-time PCR test became a gold standard in ASFV detection and is one of the recommended methods, by the World Organisation for Animal Health (WOAH, formerly OIE) [20]. However, this method may also be disturbed, i.e., by inhibitors existing in different matrices [32,33]. The presence of PCR-inhibitors or processes leading to their inactivation may result in a decreased level of detectable genetic material in samples [34]. Samples containing a higher concentration of genetic material (resulting in lower Ct in PCR-assays) are easier to interpret. In the cases of oral and rectal swabs, due to the low level of ASFV DNA copies, inhibitors and the processing of samples may result in obtaining doubtful or false-negative results. Low viral titers in oral and rectal samples have been reported before [22,24,35]. These results are in line with results presented in our study, but it should be underlined that low viral load may become an important problem during laboratory analysis. Based on the results of our and previously published studies, PCR methods are generally adequate for virulent strains of ASFV. Attenuated strains of ASFV may circulate undetected, a fact which was noticed by Gallardo et al. [36]. A low percentage of PCR-positive samples but a high antibody-mediated immune response in the group infected by attenuated NH/P68 strain proves that serological methods may be more adequate for the detection of an infection caused by attenuated ASFV strains.

Third, is the choice of an adequate time for sampling. The results of our study showed that the majority of ASFV-infected animals could be found PCR-positive when sampled regularly. However, veterinary surveillance usually relies on periodic screening, which may pose an additional risk of a missed detection of the pathogen. The percentage of detectable positive samples also depended on the incubation period of the disease; longer incubation periods resulted in higher percentages of negative samples. The percentage of positive samples increased during the fever period (up to 100% in blood). This shows that proceeding with veterinary surveillance among symptomatic animals or regular sampling gives a greater certainty of accurate diagnosis of the disease, which was further confirmed by Lamberga et al. [37]. Importantly, shedding in oral and rectal swabs was detected later than in blood, which is in accordance with previously published studies [14,23,24], also with a study published by Ramirez-Medina et al. where genetic material of ASFV was detectable earlier in blood than in nasal and oral swabs after intramuscular or oronasal infection [22]. Therefore, there is a risk that even symptomatic animals infected by virulent strains and sampled by oral or rectal swabs could potentially be diagnosed as negative for ASF.

Even though non-invasive sampling saves time and costs, it should be well defined which diseases are possible to diagnose without the risk of misdiagnosis. In the case of ASF, an implementation of non-invasive sampling (i.e., as a preliminary diagnostic tool) should be considered as doubtful. Results of our study regarding shedding, viral loads and time of detection are in accordance with most of the results published previously by other authors [7,21,22,23,24,25,31,35,38]. However, it is important to highlight the risk of the potential implementation of non-invasive sampling during ASF-surveillance. In light of this and previously published studies, recommendations made by WOAH regarding blood sampling in the ASF-surveillance are the most adequate and relevant [20]. Strategies for surveillance sampling should include the most reliable approaches for the detection of different ASFV strains. The majority of ASFV strains circulating in Europe and Asia belong to highly virulent and pathogenic isolates [39], however their evolution to moderate virulence has been noticed [12]. This may suggest that an uncontrolled and a long-lasting epidemic of ASF may evolve into an endemic and non-symptomatic disease in the future, such as in the case in Africa or Sardinia [13,40]. In light of this, serological methods will be useful for detecting ASFV-infected seropositive animals. Even though the presence of specific anti-ASFV antibodies in oral fluid and its suitability for diagnostic purposes was confirmed in the case of an ASFV-attenuated strain infection [41], recently circulating virulent strains of ASFV produce a small percentage of seropositive animals [42]. Therefore, blood sampling for PCR (whole blood) and serological investigation (sera) seem to be most reliable for the diagnosis of ASFV infection under different scenarios.

To the best of the authors’ knowledge this is the first publication describing possible diagnostic problems related to ASFV detection in non-invasive samples, based on results from experimental inoculation of pigs with ASFV strains of various virulence.

## 5. Conclusions

The results of this study showed that in animals infected by virulent strains of ASFV, the presence of ASFV genetic material in blood could be detected earlier, more frequently and in significantly larger quantities than in oral or rectal swab samples. In the case of attenuated strains, detection of the disease may require serological investigation, which implies blood sampling for the obtaining of sera. Therefore, blood sampling seems to be the most reliable for the diagnosis of ASF and stays in line with WOAH recommendations.

## Figures and Tables

**Table 1:** Experimental settings summary.

Trial (Trial Period)	Number of Animals	ASFV Strain (Genotype)	Strain Characteristics	Route of Infection	Dose per Animal
T1 (0–8 dpi)	n = 6	Arm07/CBM/c2 (II)	highly virulent	i.m.	10^3^ TCID_50_
T2 (0–21 dpi)	n = 7	Pol18_28298_O111 (II)	moderately/highly virulent	i.n.	10^3^ HAD_50_
T3 (0–28 dpi)	n = 6	NH/P68 (I)	attenuated	i.m.	10^3^ TCID_50_

i.m.—intramuscular, i.n.—intranasal.

**Table 2:** The detection of ASF DNA in relevant matrices or specific anti-ASFV antibodies in the respective trial period.

Trial	Blood	Blood Mean Ct (±SD)	Oral Swabs	Oral Swab Mean Ct (±SD)	Rectal Swabs	Rectal Swab Mean Ct (±SD)	Seropositive Animals	Maximum Antibodies Titer (log_10/_mL)
T1 (Arm07)	6/6	26.6 (±7.4)	5/6	35.1 (±2.3)	5/6	34.1 (±3.5)	1/6	4.0
T2 (Pol18)	6/7	23.7 (±5.0)	4/7	36.2 (±2.2)	7/7	34.3 (±3.3)	1/7	4.11
T3 (NH/P68)	4/6	38.3 (±1.2)	5/6	36.6 (±2.4)	3/6	38.0 (±1.4)	6/6	5.0

**Table 3:** Mean latent and incubation periods.

Trial	First Detection of Fever (Mean dpi (±SD))	First PCR-Detection in Blood (Mean dpi (±SD))	First PCR-Detection in Oral Swabs (Mean dpi (±SD))	First PCR-Detection in Rectal Swabs (Mean dpi (±SD))
T1 (Arm07)	3.3 (±0.7)	2.8 (±0.7)	4.2 (±1.2)	4.4 (±1.2)
T2 (Pol18)	8.6 (±3.6)	8.5 (±4.1)	12.0 (±2.5)	9.7 (±4.3)
T3 (NH/P68)	3.0 (±2.3)	14.0 (±4.0)	21 (±6.4)	11 (±3.1)

**Table 4:** Percentage of PCR-positive samples collected during experiments in whole trial periods.

Trial	Number of Relevant Sampling Timepoints (Blood and Swabs) in Respective Trial Periods (Trial Period)	Oral Swabs —Positive (%)	Rectal Swabs—Positive (%)	Blood—Positive (%)
T1 (Arm07)	16 (0–8 dpi)	6/16 (37.5%)	6/16 (37.5%)	11/16 (68.8%)
T2 (Pol18)	43 (0–21 dpi)	13/43 (30.2%)	16/43 (37.2%)	24/43 (55.8%)
T3 (NH/P68)	63 (0–28 dpi)	4/63 (6.3%)	3/63 (4.8%)	7/63 (11.1%)

**Table 5:** Percentage of PCR-positive samples collected during fever period.

Trial	Number of Relevant Sampling Timepoints (Blood and Swabs) during Fever	Oral Swabs—Positive (%)	Rectal Swabs—Positive (%)	Blood—Positive (%)
T1 (Arm07)	9	6/9 (66.6%)	6/9 (66.6%)	9/9 (100%)
T2 (Pol18)	20	13/20 (65.0%)	16/20 (80%)	20/20 (100%)
T3 (NH/P68)	14	0/14 (0%)	0/14 (0%)	2/14 (14.3%)

## Supplementary Materials

The following supporting information can be downloaded at: https://www.mdpi.com/article/10.3390/v14081756/s1, Figure S1: Rectal temperature and survival rate recorded during trials. Table S1: Detection of ASFV DNA in different matrices during whole trial period, individual results.
