# Supplementary material for: Non-Invasive Sampling in the Aspect of African Swine Fever Detection—A Risk to Accurate Diagnosis
Source: Viruses. 2022 Aug 11;14(8):1756. doi: 10.3390/v14081756 (PMC9416727; doi:10.3390/v14081756)
Supplement: Supplementary file 1 [file viruses-14-01756-s001.zip › viruses-1808870-supplementary.pdf]

Table S1. Detection of ASFV DNA in different matrices during whole trial period – individual results.

Trial/Trial period	Pig No	Oral	Rectal	Blood
T#1 (Arm07) 0-8dpi	#1	POS	POS	POS
	#2	POS	POS	POS
	#3	POS*	NEG	POS*
	#4	POS	POS	POS
	#5	POS	POS	POS
	#6	POS	POS*	POS
T#2 (Pol18) 0-21dpi	#1	POS	POS	POS
	#2	NEG	POS	POS
	#3	POS*	POS*	POS
	#4	NEG	POS*	POS
	#5	NEG	POS*	NEG
	#6	POS	POS	POS
	#7	POS	POS	POS
T#3 (NH/P68) 0-28dpi	#1	POS	NEG	NEG
	#2	POS	POS*	NEG
	#3	NEG	POS	POS*
	#4	POS*	NEG	POS
	#5	POS	POS*	POS
	#6	POS	NEG	POS*

*single detection, POS- positive, NEG – negative

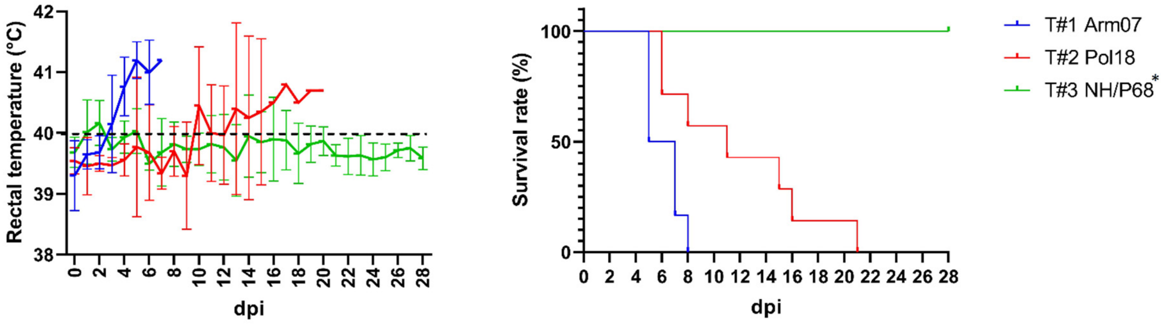

Figure S1. Rectal temperature and survival rate recorded during trials. Error bars indicate standard deviation. *-euthanized at 28 dpi
